# *ANK3* related neurodevelopmental disorders: expanding the spectrum of heterozygous loss-of-function variants

**DOI:** 10.1007/s10048-021-00655-4

**Published:** 2021-07-03

**Authors:** Katja Kloth, Bernarda Lozic, Julia Tagoe, Mariëtte J. V. Hoffer, Amelie Van der Ven, Holger Thiele, Janine Altmüller, Christian Kubisch, Ping Yee Billie Au, Jonas Denecke, Emilia K. Bijlsma, Davor Lessel

**Affiliations:** 1 Institute of Human Genetics, University Medical Center Hamburg-Eppendorf, Martinistrasse 52, 20246 Hamburg, Germany; 2 Present Address: Department of Pediatric Hematology and Oncology, University Medical Center Hamburg-Eppendorf, Hamburg, Germany; 3 Department of Pediatrics, University Hospital Split, School of Medicine, University of Split, Split, Croatia; 4 Lethbridge Outreach Genetic Services, Alberta Health Services, Lethbridge, AB T1J 1W5 Canada; 5 Department of Clinical Genetics, Leiden University Medical Center, Leiden, Netherlands; 6 Cologne Center for Genomics, University of Cologne and University Hospital Cologne, Cologne, Germany; 7 Department of Medical Genetics, Alberta Children’s Hospital Research Institute, Cumming School of Medicine, University of Calgary, Calgary, AB T2N 4N1 Canada; 8 Department of Pediatrics, University Medical Center Hamburg-Eppendorf, 20246 Hamburg, Germany

**Keywords:** ANK3, Ankyrin-G, Isoform-based phenotypic continuum, Intellectual disability, Developmental delay

## Abstract

*ANK3* encodes multiple isoforms of ankyrin-G, resulting in variegated tissue expression and function, especially regarding its role in neuronal development. Based on the zygosity, location, and type, *ANK3* variants result in different neurodevelopmental phenotypes. Autism spectrum disorder has been associated with heterozygous missense variants in *ANK3*, whereas a more severe neurodevelopmental phenotype is caused by isoform-dependent, autosomal-dominant, or autosomal-recessive loss-of-function variants. Here, we present four individuals affected by a variable neurodevelopmental phenotype harboring a heterozygous frameshift or nonsense variant affecting all *ANK3* transcripts. Thus, we provide further evidence of an isoform-based phenotypic continuum underlying *ANK3*-associated pathologies and expand its phenotypic spectrum.

**Supplementary Information:**

The online version contains supplementary material available at 10.1007/s10048-021-00655-4.

## Introduction

ANK3 codes for a large ankyrin, ankyrin-G, which is integral for spectrin-actin cytoskeleton membrane stability and scaffolding [1, 2]. It plays an important role in neuronal development, cell motility, proliferation, and signaling as well as the maintenance of specialized membrane domains and the targeting of ion channels and cell adhesion molecules [3–6]. It was initially identified in neurons in the central and peripheral nervous system, namely the nodes of Ranvier and the axon initial segment (AIS) [5, 7]. Like other members of the ankyrin family, ankyrin-G consists of four functional domains: an amino-terminal domain containing multiple ankyrin-repeats, a central spectrin-binding domain, a serine-threonine-rich neurospecific domain, and a carboxy-terminal regulatory death [8, 9].

There are multiple isoforms of ANK3 which are developmentally regulated and differentially expressed due to tissue-specific splicing [10, 11]. Notably, three major brain-specific isoforms of 190 kDa, 270 kDa, and 480 kDa (giant AnkG) have been identified, each having a unique neuronal circuit function and distribution pattern [5, 12–15]. The 190-kDA ANK3 isoform regulates dendritic spine morphology and N-methyl-D-aspartate (NMDA) receptor trafficking and is expressed almost ubiquitously in the brain [15]. The two bigger brain-specific isoforms sustain the myelin sheaths and play an important part in the formation of the nodes of Ranvier as well as the regulation of somatodendritic GABAergic synapses [12–14].

In line with its predominant neurological functions, pathogenic variants in *ANK3* have been linked to a broad range of neurological diseases, such as autism spectrum disorder (ASD), schizophrenia, bipolar disorder, and, more recently, intellectual disability (ID), epileptic encephalopathy, and behavioral abnormalities including a sleep and/or attention-deficit hyperactivity disorder (ADHD) [1, 9, 16–21]. In 2017, we reported the first individual harboring a heterozygous de novo nonsense variant which affected all three *ANK3* isoforms who presented with mild intellectual disability, autistic, and behavioral abnormalities [21]. We and others [22] postulated an isoform-specific phenotypic continuum for missense and truncating variants affecting different brain-specific isoforms of *ANK3*, resulting in an array of neurological features depending on the zygosity, location and type of variant, and the isoform affected [21].

Here, we present four novel heterozygous loss-of-function (LOF) variants affecting all isoforms of *ANK3*. The highly variable severity of cognitive impairment and other symptoms in these patients further backs the claim of an isoform-based genotypic-phenotypic continuum for *ANK3*-associated neuropathies.

## Materials and methods

### Patient information

All biological samples were obtained following written informed consent from studied individuals or their legal representatives. The study was performed in accordance with protocols approved by the Ethics Committee of the Hamburg Chamber of Physicians (PV 3802). The study was performed in accordance with the Declaration of Helsinki protocols.

### Genetic analyses

Genomic DNA was isolated from whole blood samples using standard procedures. Whole-exome sequencing (WES) in individuals 1 and 3, and trio whole-exome sequencing (trio-WES) in individuals 2 and 4, data annotation, and interpretation were performed using methods that were described previously [23–25].

## Results

### Patient characteristics

Proband 1 is the first child of non-related, healthy Caucasian parents. He was born at term after an uneventful pregnancy with a slightly reduced birth weight 2790 g (3rd percentile, − 1.89z), length 50 cm (− 1.09z), and occipitofrontal head circumference (OFC) 34 cm (− 1.23z), APGAR 10/10. At 2 years, speech delay was diagnosed. Motor development and brain MRI were unremarkable. At 5 years, the last clinical examination revealed intellectual disability (SON-IQ 55), speech delay (level of speech at 4.2 years at least 2 years delayed), and suspicion of ASD (not formally tested). His last body measurements were within the normal range; he showed no signs of seizures, muscular hypotonia, or spasticity. Facial dysmorphism was not reported.

**Fig. 1:**
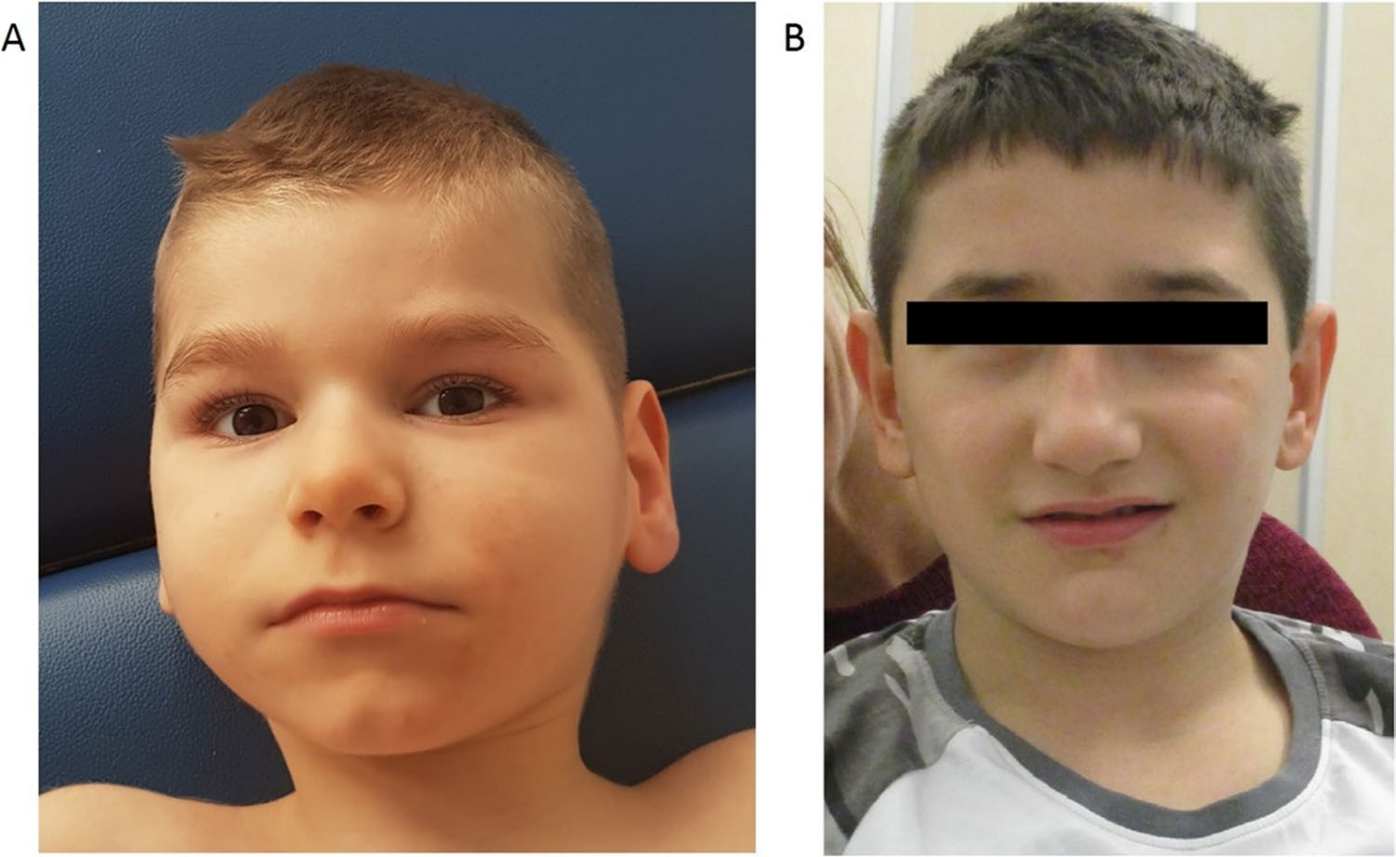
Photographs of the probands 3 and 4. **a** Frontal photographs of proband 3: mildly triangular face, small chin, and prominent lower lip vermilion; **b** frontal photographs of proband 4: short philtrum and thin upper lip vermillion

Proband 2 is the first and only child of non-related, healthy Caucasian parents. He was born after an uneventful pregnancy at 39 weeks of gestation with unremarkable birth weight 3290 g (− 0.45z), length 50 cm (− 0.83z), and OFC 34 cm (− 1z), APGAR 10/10. Developmental delay became evident at 6 months. Seizures initially presented as infantile spasms, with a diagnosis of West syndrome subsequently made due hypsarrhythmia in interictal EEG. Brain MRI revealed anomalies consistent with a type 1 lissencephaly. Swallowing and breathing difficulties were accompanied by recurrent pneumonia, and he required a percutaneous endoscopic gastrostomy (PEG). At 7 years (Fig. 1a), the most recent clinical examination revealed severe global developmental delay, lack of gross, and fine motor development with involuntary repetitive stereotype movements (rotation of the whole body; flexion–extension movement of the limbs), severe axial hypotonia (no head control, nor sitting or any controlled movement), cognitive impairment, absent social eye contact, divergent strabismus, and absent speech. Seizures were recurring and EEG remained abnormal. His latest measurements showed progressive, secondary microcephaly, and mild dystrophy (head circumference 47.5 cm (− 3.88z), weight 18 kg (− 2.27z), height 117 cm (− 1.38z)). Facial dysmorphism was not reported. Fragile X and array-CGH analyses yielded normal results.

Proband 3 is the first child of non-related, healthy Caucasian parents. Her father passed away in an accident. She has a full sibling and two maternal half siblings who have no learning difficulties. She was born after an uneventful pregnancy at 38 weeks gestation with normal measurements. Meconium was present at delivery, APGAR 6/7. She required a 3-day NICU admission, treated for presumed infection due to apneas, poor feeding, and unstable temperature. She presented with mildly delayed motor development (unaided walking at 18 months). Although first words reportedly occurred around the first year, due to significant speech delay she received speech therapy in preschool. In kindergarten she struggled with completing tasks and understanding concepts and was subsequently home-schooled. At 13 years psychoeducational testing showed a FSIQ of 68. Autistic features were not noted but she is shy and quiet. At 14 years, she was operating at a grade 2–3 level. She had a compound melanocytic nevus removed at age 11 years. There were no vision or hearing concerns, no history of seizures nor gait abnormality or stereotypic movements. Puberty was normal with menses starting at 12 years. Her latest clinical examination at 14 years showed an increased head circumference at 58 cm (+ 2.6z) and normal weight 66.15 kg (+ 1.1z) and height 169.3 cm (+ 1z). A mildly triangular face, small chin and prominent lower lip vermilion were noted (see Fig. 1a). Chromosomal microarray and fragile X testing were unremarkable.

Proband 4 is the second child of non-related, healthy Caucasian parents. His elder brother had mild speech difficulties and behavioral problems; genetic testing for the familial *ANK3* variant was unremarkable (additional patient characteristics are given in Online Resource 1). Two paternal cousins were thought to have developmental delay, however, contact was limited and no further information could be obtained. Proband 4 was born after an uneventful pregnancy at 35 weeks of gestation. Birth measurements were unremarkable; APGAR 8/9. He initially required a feeding tube; cerebral ultrasound indicated a low-grade intracerebral hemorrhage which was not detectable in later examinations. Delayed motor development was noted at 4 months, and motor milestones continued to be delayed throughout childhood (e.g. supported sitting with 12 months, unassisted walking with 3 years). At 11 years he could run and use a tricycle (no swimming, no bicycle). Expressive and receptive language skills were profoundly impaired (active language of ~ 10 words) and he communicated using a language computer. Slow but continuous progress regarding verbal and motor development without signs of regression was reported. He attended special school, as writing and reading skills had not been achieved. Social interaction, sleeping and eating behavior were unremarkable. However, he displayed repetitive stereotypic movements. At the latest examination his weight was 36.6 kg (+ 0.1z), body length was 131 cm (− 1.9z) and OFC was 52 cm (− 1.4z). Hearing and ophthalmologic examination, extensive laboratory work-up (including endocrine and metabolic analyses) gave normal results. His EEGs showed markers for an increased susceptibility to seizures. Brain MRI detected multiple flair hyperintensities of the white matter periventricular and hypoplastic corpus callosum. Facially, a short philtrum and thin upper lip vermillion were noted (see Fig. 1b).

### WES analyses

**Fig. 2:**
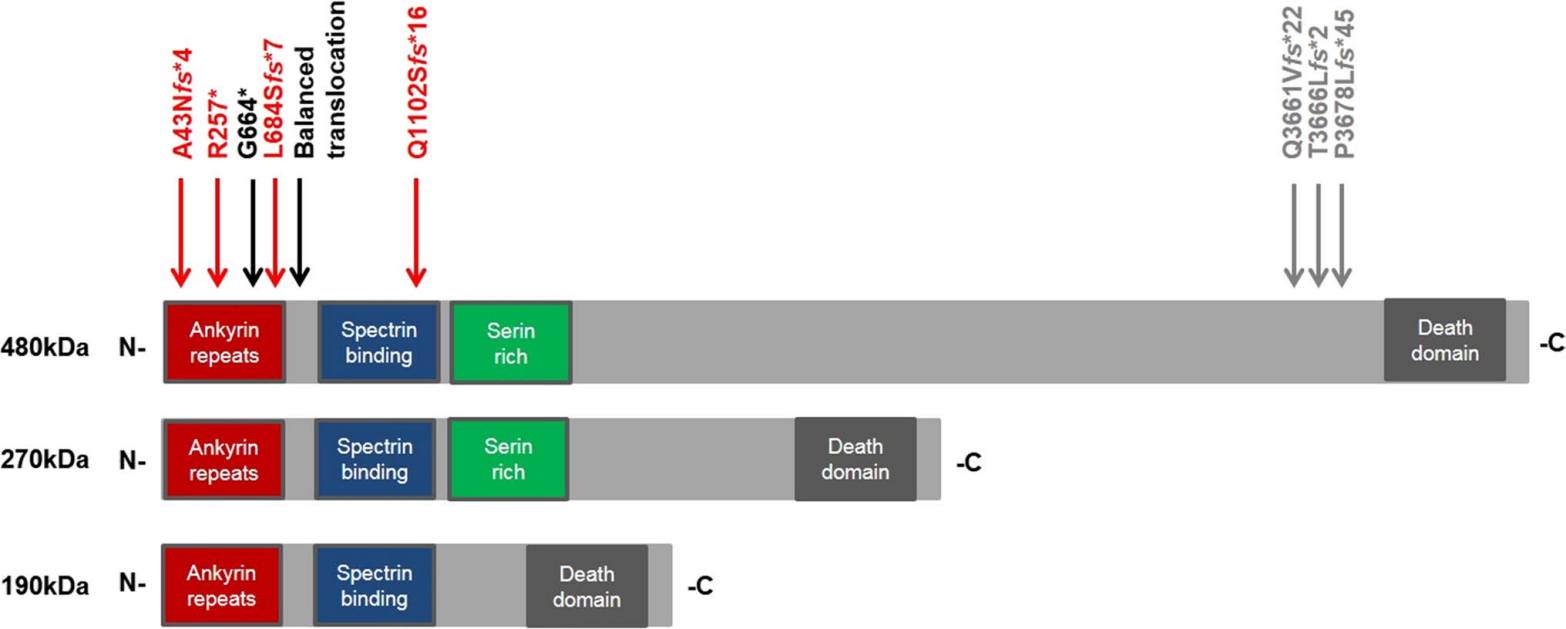
Protein structure of the three *ANK3* isoforms with indicated positions of identified loss-of-function variants (as previously described by Kloth et al., 2017). Red arrows indicate position of the here identified heterozygous variants; black arrows indicate position of the previously reported variants affecting all three *ANK3* isoforms and gray arrows indicate previously reported homozygous variants affecting only a single *ANK3* isoform

Duo-WES with samples of the proband 1 and his mother revealed a heterozygous 1 bp-deletion in *ANK3*, c.2050delG, p.(Leu684Serfs*7) that was absent in the mother. Trio-WES analysis in probands 2 and 4 revealed in each a novel de novo heterozygous 1 bp-deletion in *ANK3*, c.127delG and c.3303delC, p.(Ala43Glnfs*4) and p.(Glu1102Serfs*16), respectively. Duo-WES in proband 3 and her mother revealed a heterozygous single-nucleotide variant in *ANK3*, c.769C > T, p.(Arg257*), which was not maternally inherited. Paternal DNA samples of probands 1 and 3 were unavailable for testing. None of these variants were present in the gnomAD dataset v2.1.1. All four variants affected all three *ANK3* isoforms (Fig. 2) and likely activate nonsense-mediated mRNA decay (NMD).

## Discussion

Different types of variants affecting in part different isoforms of the *ANK3* gene have been associated with a wide array of neurological pathologies [1, 9, 17, 18, 20–22]. A genotypic-phenotypic continuum for missense and nonsense mutations in *ANK3* has been proposed and potentially offers an explanation for the highly variable severity of the neurological and neurodevelopmental symptoms [22]. Autosomal recessive missense variants that disturb the transition of giant AnkG from a closed to an open conformation have been identified in two individuals affected by a varied spectrum of neurodevelopmental clinical signs and symptoms [9]. Similar clinical outcome was observed in cases harboring homozygous frameshift variants that also affect only the largest 480-kDa isoform [22]. Moreover, heterozygous missense variants have been associated with a milder clinical spectrum ranging from ASD to neuropsychiatric disorders like schizophrenia and bipolar disorder [1, 16–18]. In addition, heterozygous loss-of-function (LOF) variants affecting all brain-specific isoforms of *ANK3* have been identified in individuals affected by a neurodevelopmental disorder [21, 22]. Here we report four additional unrelated individuals harboring heterozygous LOF variants affecting all three major brain *ANK3* isoforms. Interestingly, they display a varied spectrum of neurodevelopmental phenotypes (see Table 1).

**Table 1:** Clinical features of affected individuals harboring loss-of-function variants affecting all three *ANK3* isoforms (+ , present; -, absent; *n/a*, unknown; *y*, years)

Affected individuals	Individual 1	Individual 2	Individual 3	Individual 4	Previously described individual (Kloth et al., 2017)	Previously described individual (Iqbal et al., 2013)
Sex	Male	Male	Female	Male	Male	Male
Genotype	Heterozygous	Heterozygous	Heterozygous	Heterozygous	Heterozygous	Heterozygous
	Not present in the mother, father n/a	De novo	Not present in the mother, father n/a	De novo	De novo	n/a
	1 bp deletion	1 bp deletion	Nonsense	1 bp deletion	Nonsense	Translocation
Age at last examination	5 y	7 y	14 y	11 y	9 y	14 y
Intellectual disability	+ IQ at the age of 4.5 y: SON-IQ 55	+	+ IQ at age 13 y: FSIQ 68	+	+ (mild)	+ (mild)
Speech delay	+ (severe)	+ (absent speech)	+ (significant)	+	+ (mild)	+
Muscular hypotonia	-	+ (severe, no head control)	-	+	+	+
Spasticity	-	+	-	-	-	-
Stereotypic limb movements	-	+	-	+	-	- (repetitive behavior)
Hyperactivity	-	-	-	-	+	+
Autistic features	+ (not formally diagnosed)	+	- (described as shy and quiet)	-	+	+
Epilepsy	-	+ (onset 6 m)	-	- (susceptibility to seizures on EEG)	-	-
Aggressiveness	-	-	-	-	+	+
Sleeping disorder	-	-	-	-	+	+
Chronic hunger	-	-	- Poor feeding in infancy	-	+	n/a
Dysmorphic features	-	-	( +) Slightly triangular face, prominent lower lip vermilion, small chin	( +) Short philtrum, thin upper lip vermillion	-	( +) Flat philtrum, broad nose, upslanted palpebral fissures
Macrocephaly	-	- (microcephaly)	+	-	+	- (bordering microcephaly)
Macrosomia	-	- (feeding difficulties in infancy)	-	- (feeding difficulties in infancy)	+	n/a
cCT/brain MRI	Not done	Type 1 lissencephaly	Not done	Multiple flair hyperintensities within the white matter, periventricular, parietal, near the insula, hypoplastic corpus callosum	Normal	n/a
*ANK3* variant	c.2050delG; p.(Leu684Serfs*7)	c.127delG; p.(Ala43Glnfs*4)	c.769C > T, p.(Arg257*)	c.3303delC; p.(Glu1102Serfs*16)	c.1990G > T; p.(Gly664*)	Balanced translocation

All four presented with intellectual disability and speech delay, similar to two previously reported cases bearing similar *ANK3* loss-of-function variants [21, 22]. Autistic features and delay in motor development, at least mild, were observed in three individuals. Muscular hypotonia was present in two individuals, in one of them severe. Brain MRI was conducted in two individuals: it showed type 1 lissencephaly in the most severely affected proband 2, whereas proband 4 presented with multiple white matter hyperintensities and hypoplastic corpus callosum. Epilepsy was diagnosed in proband 2 (West syndrome), whereas proband 4 showed markers for a susceptibility to seizures on EEG without clinical seizures. Macrocephaly that we previously reported [21] was described only in proband 3, while the lissencephalic patient developed secondary microcephaly; the two other probands presented with unremarkable OFCs.

Noteworthy, proband 2 presented with the most severe clinical course of all so far known individuals harboring a LOF variant. Interestingly, p.(Ala43Glnfs*4) is the most N-terminally located LOF variant that has been identified so far, and performed trio-WES analysis detected no other likely pathogenic variants. This variant will most likely lead to haploinsufficiency, representing a fully non-functional protein or null-allele. One possibility for the more severe clinical course of this individual as compared to the other ones presented here or previously [21, 22], could be that the more C-terminal nonsense and frameshift variants still result in a stable but truncated protein which does retain some of the ANK3 function. Clearly, western blot analyses utilizing proband-derived primary material, which was unfortunately not available here, are needed to clarify this. It is also worth noting that we cannot completely exclude the possibility that proband 2 harbors a further distinct genetic factor that has not been covered by WES.

However, as brain anomalies were detected in proband 4 and proband 3 had low IQ and macrocephaly, suggesting brain anomalies although brain imaging was not conducted, structural brain anomalies may represent an expansion of the clinical spectrum of *ANK3*‐related disorders. Thus, it might be warranted to perform singular brain scan in every individual harboring a likely pathogenic *ANK3* variant and to consider *ANK3* analysis in undiagnosed cases with structural brain anomalies. Additionally, two of the four probands presented with repetitive stereotypic movements. Since a stereotypic movement disorder (SMD) was not formally diagnosed, it remains difficult to differentiate between SMD and stereotypic autistic behavior—but stereotypic limb movements might represent a novel *ANK3*-related symptom. Notably, aggressiveness, hyperactivity, signs of a sleeping disorder, or chronic hunger remain unique to the individual we reported previously [21]. In line with our previous report, there were no recognizable dysmorphic features in this cohort; although mild variations of the lips, philtrum and/or chin area were described in two individuals (see Fig. 1a + b). Thus, as of now, we would not reliably consider these features part *ANK3*-associated phenotype. Further clinical reports beyond the probands identified in this study are needed to elucidate the full phenotypic spectrum and broaden genotype–phenotype correlations. The latter especially holds true for the occurrence and degree of speech delay, intellectual disability, neuromuscular involvement, structural brain anomalies, epilepsy, and behavioral abnormalities.

Taken together, this cohort allows an expansion of the *ANK3*-mediated clinical spectrum and adds further evidence to the existence of an autosomal dominant *ANK3*-related neurodevelopmental disorder as well as an isoform-based, phenotypic continuum between dominant and recessive *ANK3*-associated pathologies.

## Supplementary Information

Below is the link to the electronic supplementary material.

Supplementary file1 (DOCX 15 KB)
