# Supplementary material for: ANK3 related neurodevelopmental disorders: expanding the spectrum of heterozygous loss-of-function variants
Source: Neurogenetics. 2021 Jul 3;22(4):263–9. doi: 10.1007/s10048-021-00655-4 (PMC8426245; doi:10.1007/s10048-021-00655-4)
Supplement: Supplementary file 1 — Supplementary file1 (DOCX 15 KB) [file 10048_2021_655_MOESM1_ESM.docx]

**Supplementary Information (SI)**

**Online Resource 1) Patient characteristics of brother of Proband 3**

The elder brother of Proband 3 was born after an uneventful pregnancy with unremarkable birth measurements. Psychomotor development in the first year was reportedly normal. He spoke first words before 12 months; however, shortly after his first birthday, he abruptly discontinued speaking. He resumed active language around the age of 4.5 years. Cognitive and motor development was described age-appropriate throughout childhood. He acquired reading, writing and math skills at an appropriate age. At the age of 8 years, he was enrolled in a specialized, integrative class of regular school but soon had to be transferred to special schooling. He displays features of autistic behavior (e.g. ritualized daily routine, need for order and rules); no formal ASD testing was conducted. Social behavior was otherwise described to be normal, without indications of aggression or hyperactivity. Sleeping and eating was reportedly normal. His body measurements were age-appropriate. A previously performed EEG was reported unremarkable; brain MRI has not been conducted. Direct Sanger sequencing excluded the *ANK3* variant identified in proband 3,
